# Is It Safe to Do Percutaneous Coronary Intervention in Moderate to Severe Chronic Kidney Disease Patients? A Prospective Cohort Study

**DOI:** 10.7759/cureus.30312

**Published:** 2022-10-14

**Authors:** Yudistira Santosa, Azizah Dhena Harca, Angelina Yuwono, Amanda Hermanto, Muhammad S Oliver, Edwin Sukmadja, Ratna Soewardi

**Affiliations:** 1 Internal Medicine, Atma Jaya Catholic University of Indonesia, Jakarta, IDN; 2 Emergency Department, Primaya Hospital, Jakarta, IDN; 3 Emergency Department, Primaya Hospital, Banten, IDN; 4 Emergency Department, Primaya Hospital, Tangerang, IDN

**Keywords:** percutaneous coronary intervention, chronic kidney disease, contrast induced nephropathy prophylaxis, rehydration, acetylcysteine, sodium bicarbonate

## Abstract

Introduction: Contrast-induced acute kidney injury (CI-AKI) is a common and potentially serious complication of percutaneous coronary intervention (PCI) procedures, as it induces acute kidney injury (AKI), especially in previously diagnosed chronic kidney disease (CKD) patients, particularly in those who also have diabetes. Adequate hydration and using a minimal volume of contrast media are the recommended measures to decrease CI-AKI in CKD patients. A combination of sodium bicarbonate and N-acetylcysteine (NAC) may be a superior strategy for preventing CI-AKI. This study is aimed to evaluate the safety of PCI in moderate to severe CKD patients.

Method: This was a prospective, single-center study, from 2019 to 2021. We included all chronic kidney disease who undergo PCI procedures. The kidney level was measured on admission and 24 hours after the PCI procedure. The patients received 75 meq/500 mL of sodium bicarbonate one to six hours before procedures, oral acetylcysteine 600 mg bid for three days, and rehydration with 1000 ml of normal saline infusion for eight hours in patients without congestive heart failure. SPSS Version 23.0 (IBM SPSS Statistics for Windows, Version 23.0., IBM Corp., Armonk, NY) was used to input and process the data.

Results: This study included 118 subjects, with baseline characteristics of age 58.77 ± 9.08 years, 80.5% male, 47.5% diabetic, 50% hypertension, and 59.5% congestive heart failure. From the coronary angiogram, we found most of our subjects (57.6%) had three-vessel disease, 28.8% had two-vessel disease, and 15.6% had one-vessel disease. About 67.8% of subjects used <50 ml of low molecular contrast. The baseline creatinine level was 2.46 ± 1.04 mg/dL and the estimated glomerular filtration rate (eGFR) was 30 ± 12.65 mL/min. There were 19 (16.1%) patients with stage 3A CKD, 45 (38.1%) stage 3B, 41 (34.7%) stage 4, and 41 (34.7%) stage 5. The kidney function test after 24 hours of contrast admission showed a creatinine level of 2.37 ± 1.20 mg/dL (P<0.05) and the eGFR of 34.74 ± 16.10 mL/min. There was no significant difference in creatinine levels between stage 3A and stage 5 CKD. There was a significant reduction in creatinine in stage 3B CKD, from 1.917 ± 0.22 to 1.71 ± 0.37 mg/dL (P = 0.001); and stage 4 CKD, from 2.77 ± 0.55 to 2.72 ± 0.94 mg/dL (P = 0.013).

Discussion: CKD is a risk factor for developing CI-AKI after PCI, which is a marker of poor long-term outcomes. The development of CI-AKI is a strong predictor of post-PCI bleeding, which aggravates hemodynamic instability. The combination of NAC and NaHCO_3_ exerts a better antioxidative effect, which reduces the harmful short-term and long-term consequences of contrast media. Previous studies revealed the use of low-to-zero contrast media was safer in CKD patients who had undergone PCI. By applying these measures, our study showed a good outcome of PCI with no worsening renal function in CKD patients.

Conclusion: With good prophylaxis measures, such as using minimal volume contrast media, adequate rehydration, and the combination of sodium bicarbonate and acetylcysteine, it is safe to do PCI in moderate to severe CKD patients.

## Introduction

Contrast-induced acute kidney injury (CI-AKI) is a potentially life-threatening complication of percutaneous coronary intervention (PCI) procedures. According to the acute kidney injury network (AKIN), CI-AKI is diagnosed if at least one of the following criteria is met: (1) absolute increase in serum creatinine (SCr) by >0.3 mg/dL from baseline; (2) relative increase in SCr by >50% from baseline; and (3) urine output is reduced to <0.5 ml/kg/hours for at least six hours [1]. Others defined CI-AKI as over 0.5 mg/dl or >25% increase in serum creatinine 48 hours after the administration of the contrast medium, without any other cause of AKI [2].

Various studies reported that CI-AKI occurred in up to 30% of patients receiving intra-arterial contrast media [3-6]. In a previous study in Indonesia from a state hospital in Bali, 7 out of 50 patients developed CI-AKI after contrast administration [7]. Patients who undergo PCI procedures and develop CI-AKI are at higher risk of short- and long-term mortality. CI-AKI is also associated with post-PCI cardiovascular events and in-hospital events such as the need for bypass surgery, bleeding and blood transfusion, and other vascular complications [8,9]. Several factors contribute to the development of AKI due to contrast media, such as diabetes mellitus, age, congestive heart failure, and previous history of kidney disease [2]. Of all factors, the history of stage 3 to 5 chronic kidney disease (CKD) and diabetes are known to be the most powerful independent risk factors for developing contrast-induced AKI [8,10].

Iodinated contrast (tri-iodinated benzene) is used in the coronary angiography procedure. Cytotoxicity occurs after the contrast administration and the endothelial cells produce vasoactive mediators (nitrous oxide, adenosine, prostaglandin, endothelin, and reactive oxygen species (ROS)), which later cause prolonged renal vasoconstriction, impaired renal perfusion, and ischemia [11]. The renal medulla injury is the end result of ischemia due to CI-AKI [12]. Adequate preprocedural hydration, using a minimal volume of contrast media and high-intensity statin has been recommended to prevent CI-AKI in CKD patients undergoing angiography [8,13]. Bicarbonate sodium and high-dose acetylcysteine have been studied as CI-AKI prevention, although it is not recommended in routine practices, some studies showed the benefit of CI-AKI prevention [14,15]. In this study, we aim to evaluate renal function 24 hours after administering contrast media for PCI in CKD patients, who were also given hydration, minimal volume contrast media, bicarbonate sodium, and acetylcysteine.

## Materials and methods

Study population

This was a single-center, prospective cohort study, which was conducted at Primaya Hospital, Tangerang City, Banten, Indonesia. Study participants were taken using the consecutive sampling method. We included patients over 18 years old who had a history of CKD stage 3 or over, a history of coronary arterial disease (CAD) (stable), underwent the PCI procedure and were checked for serum creatinine level on admission and 24 hours after the procedure. Patients with unstable hemodynamics were excluded from the trial.

Study protocol

All patients received 75 meq/500 ml bicarbonate sodium one to six hours before procedures, oral acetylcysteine 600 mg bid for three days, and rehydration with 1000 ml for eight hours (given two hours pre-contrast and six hours after contrast). Kidney function measurement was assessed on admission and 24 hours after PCI procedures to see any kidney function decline within the first 24 hours. This study was approved by the ethics committee of Primaya Hospital Tangerang (previously known as Awal Bros Hospital), with reference number 001/KOMED-EXT/RSABT/IX/2019.

Data collection and definition of variable

The patients were classified by sex (male and female), a history of previously diagnosed hypertension, diabetes mellitus, and heart failure was obtained. The contrast volume used was classified as less than 50 ml or over 50 ml. From the coronary angiogram, we described the number of vessels affected (1/2/3 vessel disease). We used the 2012 Kidney Disease Improving Global Outcomes (KDIGO) CKD classification to group the CKD patients, whereas CKD is defined as a function or structural abnormalities of the kidney presented at least three months. Glomerular filtration rate (GFR) is used for the classification of CKD, of which over 90 ml/min/1.73 m^2^ is classified as stage 1, 60-90 ml/min/1.73 m^2^ is classified as stage 2, 45-59 ml/min/1.73 m^2^ is classified as stage 3A, 30-44 ml/min/1.73 m^2^ is classified as stage 3B, 15-29 ml/min/1.73 m^2^ is classified as stage 4, and less than 15 ml/min/1.73 m^2^ is stage 5 [16]. The kidney function is measured as serum creatinine (mg/dL). All data is input and processed with SPSS Version 23.0 (IBM SPSS Statistics for Windows, Version 23.0., IBM Corp., Armonk, NY). We used a dependent t-test to evaluate serum creatinine changes pre- and post-contrast.

## Results

This study included 118 subjects, with baseline characteristics (Table 1) of age 58.77 ± 9.08 years, 80.5% male, 47.5% diabetic, 50% hypertension, and 59.5% congestive heart failure. From the coronary angiogram, we found most of our subjects (57.6%) had three-vessel disease, 28.8% had two-vessel disease, and 15.6% had one-vessel disease. About 67.8% of subjects used <50 ml of low molecular contrast. The baseline creatinine level was 2.46 ± 1.04 mg/dL and the estimated glomerular filtration rate (eGFR) was 30 ± 12.65 mL/min. There were 19 (16.1%) patients with stage 3A CKD, 45 (38.1%) with stage 3B, 41 (34.7%) with stage 4, and 41 (34.7%) with stage 5. The kidney function test after 24 hours of contrast admission showed a creatinine level of 2.37 ± 1.20 mg/dL (P<0.05) and the eGFR of 34.74 ± 16.10 mL/min. There was no significant difference in creatinine levels between stage 3A and stage 5 CKD. There was a significant reduction in creatinine in stage 3B CKD, from 1.917 ± 0.22 to 1.71 ± 0.37 mg/dL (P = 0.001); and stage 4 CKD, from 2.77 ± 0.55 to 2.72 ± 0.94 mg/dL (P = 0.013). The comparison of kidney function can be seen in Table 2.

**Table 1:** Baseline characteristics of the subjects.

Variables	Total patients (%) n = 118	Mean (±SD)
Age (years)		58.77 ± 9.08
Sex		
Male	95 (80.5%)	
Female	23 (19.5%)	
Hypertension		
Yes	59 (50%)	
No	59 (50%)	
Type 2 diabetes mellitus		
Yes	56 (47.5%)	
No	62 (52.5%)	
Heart failure		
Yes	70 (59.3%)	
No	48 (40.7%)	
Volume contrast		
Below 50 cc	80 (67.8%)	
Above 50 cc	38 (32.2%)	
Angiogram		
One vessel disease	16 (13.6%)	
Two vessel disease	34 (28.8%)	
Three vessel disease	68 (57.6%)	
Stages of chronic kidney disease		
Stage 3A	19 (16.1%)	
Stage 3B	45 (38.1%)	
Stage 4	41 (34.7%)	
Stage 5	13 (11%)	

**Table 2:** Kidney function pre-PCI and post-PCI. PCI: percutaneous coronary intervention; eGFR: estimated glomerular filtration rate.

Variables	Total patients	Kidney function	P-value
Pre-PCI creatinine (mg/dl)		2.46 ± 1.04	P = 0.000157
Post-PCI creatinine (mg/dl)		2.37 ± 1.20	
Pre-PCI eGFR (ml/min/1.73 m^2^)		30.90 ± 12.65	P = 0.000003
Post-PCI eGFR (ml/min/1.73 m^2^)		34.74 ± 16.10	
Stage 3A	19 (16.1%)		
Pre-PCI creatinine (mg/dl)		1.56 ± 0.14	
Post-PCI creatinine (mg/dl)		1.55 ± 0.32	0.0387
Stage 3B	45 (38.1%)		
Pre-PCI creatinine (mg/dl)		1.917 ± 0.22	
Post-PCI creatinine (mg/dl)		1.71 ± 0.37	0.001
Stage 4	41 (34.7%)		
Pre-PCI creatinine (mg/dl)		2.77 ± 0.55	
Post-PCI creatinine (mg/dl)		2.72 ± 0.94	0.013
Stage 5	13 (11%)		
Pre-PCI creatinine (mg/dl)		4.73 ± 1.05	
Post-PCI creatinine (mg/dl)		4.73 ± 1.21	0.753

## Discussion

CKD, especially stages 4 and 5, causes a chronic and systemic proinflammatory state that results in the formation of atherosclerotic lesions, vascular calcification, vascular senescence, myocardial fibrosis, and cardiac valve calcification. This increases the prevalence of cardiovascular events, which is the leading cause of death in CKD patients [17]. Coronary angiography remains as the gold standard for diagnosing coronary arterial disease (CAD) [14]. Iodinated contrast medium given intravenously during the procedure is a common precipitator of CI-AKI [18].

Nevertheless, the risk of CI-AKI precipitating AKI and the need for dialysis in CKD patients should be weighed against the benefit of the diagnostic test. The ISCHEMIA-CKD trial, which consisted of 777 CKD subjects, revealed a significantly higher incidence of stroke, death, and initiation of dialysis in subjects who received invasive strategies (coronary angiography and revascularization) [19]. Both medical and invasive treatment risks and benefits bring a dilemma in daily practices and lead into “therapeutic nihilism” in many CKD subjects [19,20]. The previous study showed CKD patients have more severe CAD than patients with normal kidney function. The study revealed that CKD patients were often found to have three-vessel or left main disease, and the severity of CAD was proportional to the degree of CKD [21]. Our study also showed most of our CKD subjects had three-vessel disease (57.6%).

The usage of low or minimal contrast volume, adequate hydration, and the use of high-dose statins have been recommended in CI-AKI prevention. Creatinine monitoring should be done once daily for the first five days after the injection of the contrast medium [22]. A previous study showed a small volume of contrast medium (30 ml) was still able to trigger AKI in high-risk patients [23]. Some suggested giving the volume not more than twice the patient’s baseline eGFR or adjusting to the patient's body weight [23]. In this study, the majority of patients (67.8%) received less than 50 ml of contrast media. The development of nonionic low osmolar contrast media (LOCM) or nonionic iso-osmolar contrast media (IOCM) also decreases the risk of CI-AKI in patients with pre-existing kidney disease [24].

Adequate hydration should be ensured in all patients undergoing coronary angiography because it prevents CI-AKI [8,14]. The renin-angiotensin system and vasopressin are inhibited by intravenous hydration. Hydration also increases the urinary flow rate, which reduces the concentration and the transit time of contrast in the renal tubules, thus reducing the risk of direct exposure between the renal tubules and contrast media [25]. For the prevention of CI-AKI in patients with kidney disease, it is recommended to give hydration with a normal saline infusion at the rate of 1 ml/kg/hour over 6-12 hours pre-procedure and it should be continued for 24 hours after the procedure [26,27]. Some randomized control trials and meta-analyses compared the effectiveness between intravenous fluid and oral fluid, and studies revealed that oral hydration is as effective as IV saline [26]. Hydration should be done carefully in patients with severe renal insufficiency and cardiac failure due to the risk of volume overload [12]. In patients with left ventricular ejection fraction less than 40% or severe congestive heart failure, the rate of hydration should be lower to 0.5 ml/kg/hour, which should be initiated immediately before or at the emergent procedure, and continued for 4-24 hours after the procedure [25].

Intravenous bicarbonate sodium and acetylcysteine, as we gave to our subjects, have been in debate. Theoretically, bicarbonate sodium decreases urine acidification (alkalinizing effect) on the renal tubule and it may help reduce free radical injury [28]. A previous randomized controlled study by Merten et al., whose study protocol included IV hydration with sodium bicarbonate at 3 ml/kg/hour one hour before the procedure and 1 ml/kg/hour for six hours after the procedure, revealed a decrease in CI-AKI events in the sodium bicarbonate group compared with the normal saline only group, and it was statistically significant [12,28]. Another study conversely showed that there was increased CI-AKI in the sodium bicarbonate group [12]. Hagikura et al. demonstrated there was no protective effect of intravenous sodium bicarbonate, but the normal saline group showed protective effects [29]. N-acetylcysteine (NAC) has been known as a potent antioxidant and may prevent direct oxidative kidney damage [28]. Although some studies demonstrated there was no significant risk reduction by giving NAC, a study by Biguori et al. and Tepei et al. showed an improvement in serum creatinine levels in patients who were given 600 mg twice-daily oral NAC for CI-AKI prophylaxis [30]. Although if given by themselves, studies revealed there was no significant reduction, a study by Thayssen et al. showed that a combination of NAC and sodium bicarbonate gave a significant reduction of serum creatinine at 30 days of follow-up [28]. A previous meta-analysis showed that in CKD patients, a combination of NAC and sodium bicarbonate was associated with a lower CI-AKI than in those who received standard treatment [28]. Thayssen et al. revealed a significant reduction in in-hospital death and CI-AKI incidence from 14.1% to 8.0% in the NAC+NaHCO_3_ group 2 days after the contrast exposure [28].

Our subjects had been given a limited volume of contrast media, received intravenous normal saline for hydration pre- and post-procedure, and also received both intravenous bicarbonate sodium and N-acetylcysteine, which have antioxidative effects. In our study, we found there was no worsening serum creatinine in the first 24 hours after the procedure in the CKD patients, but further study should be done assessing the 48 hours renal function. We suggest comparing the first 24 hours and 48 hours post-contrast serum creatinine to see if the first 24 hours' creatinine level may predict the incidence of CI-AKI. This study was not compared between CKD patients and non-CKD patients because we aimed to see if these prevention measures may be an option for CKD patients to prevent CI-AKI. The limitation of this study is that we did not assess the daily serum creatinine, as per the previous study protocols, to assess the decline in renal function per day. We also did not compare the outcome of each comorbidity with the effect of CI-AKI prevention.

## Conclusions

With good prophylaxis measures, such as using minimal volume contrast media, adequate rehydration, and the combination of sodium bicarbonate and acetylcysteine, worsening renal function was not found in the first 24 hours in CKD patients. Further studies with larger samples and assessing the 48 hours serum creatinine and the long-term outcome of kidney function with these measures of prevention should be done.
